# Novel Mechanistic Insights and Potential Therapeutic Impact of TRPC6 in Neurovascular Coupling and Ischemic Stroke

**DOI:** 10.3390/ijms22042074

**Published:** 2021-02-19

**Authors:** Shashank Shekhar, Yedan Liu, Shaoxun Wang, Huawei Zhang, Xing Fang, Jin Zhang, Letao Fan, Baoying Zheng, Richard J. Roman, Zhen Wang, Fan Fan, George W. Booz

**Affiliations:** 1 Department of Neurology, University of Mississippi Medical Center, Jackson, MS 39216, USA; 2 Department of Pharmacology and Toxicology, University of Mississippi Medical Center, Jackson, MS 39216, USA; liuyedan1010@163.com (Y.L.); chris.wang@ucsf.edu (S.W.); zhanghw.howard@gmail.com (H.Z.); xfang@umc.edu (X.F.); alexander600229@gmail.com (J.Z.); lfan@umc.edu (L.F.); bzheng@umc.edu (B.Z.); rroman@umc.edu (R.J.R.); ffan@umc.edu (F.F.); gbooz@umc.edu (G.W.B.); 3 School of Medicine, I.M. Sechenov First Moscow State Medical University, Moscow 119048, Russia; 4 Department of Physiology and Biophysics, University of Mississippi Medical Center, Jackson, MS 39216, USA; zwang3@umc.edu

**Keywords:** blood–brain barrier, transient receptor potential cation channels, ischemic stroke, neuroprotection, calcium signaling, cAMP response element-binding protein

## Abstract

Ischemic stroke is one of the most disabling diseases and a leading cause of death globally. Despite advances in medical care, the global burden of stroke continues to grow, as no effective treatments to limit or reverse ischemic injury to the brain are available. However, recent preclinical findings have revealed the potential role of transient receptor potential cation 6 (TRPC6) channels as endogenous protectors of neuronal tissue. Activating TRPC6 in various cerebral ischemia models has been found to prevent neuronal death, whereas blocking TRPC6 enhances sensitivity to ischemia. Evidence has shown that Ca^2+^ influx through TRPC6 activates the cAMP (adenosine 3’,5’-cyclic monophosphate) response element-binding protein (CREB), an important transcription factor linked to neuronal survival. Additionally, TRPC6 activation may counter excitotoxic damage resulting from glutamate release by attenuating the activity of N-methyl-d-aspartate (NMDA) receptors of neurons by posttranslational means. Unresolved though, are the roles of TRPC6 channels in non-neuronal cells, such as astrocytes and endothelial cells. Moreover, TRPC6 channels may have detrimental effects on the blood–brain barrier, although their exact role in neurovascular coupling requires further investigation. This review discusses evidence-based cell-specific aspects of TRPC6 in the brain to assess the potential targets for ischemic stroke management.

## 1. Introduction

In the USA, someone experiences a stroke every 40 s (https://www.cdc.gov/stroke/facts.htm (accessed on 2 January 2021)) or dies of a stroke every 4 min [1]. More than 795,000 people have a stroke every year, with 77% of these being first-time strokes [1]. In 2018, one of every six deaths in America from cardiovascular disease resulted from a stroke, making it the fifth leading cause of death in the USA. Some 87% of all strokes are ischemic strokes, in which blood flow to the brain is blocked, with the remainder classified as hemorrhagic due to rupture of a weakened blood vessel [1]. Moreover, stroke-related costs involving medicines, health care, and lost employment are staggering, totaling almost USD 50 billion between 2014 and 2015 [1].

Stroke is a leading cause of severe long-term disability, reducing mobility in more than half of the survivors 65 years old and over. Stroke is also the second leading cause of death globally [2]. Although the risk of stroke increases with age, it can occur at any age and in 2009, 34% of those hospitalized for stroke were younger than the age of 65 [3]. Despite advances in medical care, the global burden of stroke continues to grow [4]. Current thrombolytic therapy works for treating ischemic stroke, but only in a limited timeframe. No drugs are approved that enhance recovery, and thus, there is a great need to identify viable pharmacological targets. Furthermore, the current COVID-19 pandemic is directly increasing the incidence of ischemic injury and is worsening the incidence and prevalence of stroke in high-risk populations, as a result—in part—of increases in sedentary lifestyles associated with social distancing [5,6].

Stroke has two major types: hemorrhagic and ischemic, with the latter caused by cerebral embolism or thrombosis [7,8]. Despite varied etiologies for ischemia and hemorrhage, oxygen deprivation to neuronal tissue is a common mechanism. With hemorrhagic stroke, which is generally more severe [9], additional damage results from irritation and swelling from pressure build-up in surrounding tissues due to bleeding and blood breakdown products. In ischemic stroke, there is sudden occlusion of cerebral blood vessels, which leads to the interruption of, or reductions in blood supply to the brain tissue, resulting in extensive neuronal death [10]. This process involves a complex cascade of events at both the macro and microscopic levels, involving impaired vascular autoregulation, disruption of the blood–brain barrier (BBB), calcium overload-associated apoptosis, and neuronal death [11,12,13]. A key step is energy depletion from reduced blood flow, leading to Na^+^/K^+^ ATPase (sodium pump) failure, which causes cell membrane depolarization and glutamate release. Na^+^/K^+^ ATPase failure results in activation of proteases, kinases, and lipases, which contribute to tissue damage and necrosis. There is also a surge in phospholipase A2 activity that results in arachidonic acid (AA) release and enhanced free radical formation and lipid peroxidation [14,15]. Combined with non-neuronal (i.e., glial) cell activation, the neurovascular unit, consisting of astrocytes, endothelial cells and their BBB forming tight junctions, and pericytes, is impaired [16]. BBB disruption is further enhanced by neuronal glutamine release that activates endothelial N-methyl-d-aspartate (NMDA) receptor-mediated intracellular Ca^2+^ influx [17,18]. Glutamine is a major contributor to Ca^2+^ influx in neurons after ischemic stroke, and thus, also to the associated neurotoxicity.

Ischemic events may additionally activate Ca^2+^ influx via a non-glutamate pathway, including via transient receptor potential cation/canonical (TRPC) channels [19,20]. Increased cytosolic Ca^2+^ with ischemia can induce apoptosis and neuronal death by several means, including activation of calpains [11,19]. TRPC channels have been linked to vasospasm in hemorrhagic stroke [21]. Paradoxically one family member, TRPC6, has been linked to neuroprotection with ischemic stroke. It is reported that activating TRPC6 in a rat model of cerebral ischemia was shown to prevent neuronal death, whereas blocking TRPC6 enhanced sensitivity to ischemia [22]. One mechanism is that in patients treated for acute ischemic stroke, elevated expression levels in the peripheral blood of miR-488 and miR-135b, which were shown to target the *TRPC6* gene, were identified as risk factors or associated with disease severity [23,24]. Here, we assess the evidence for and against the beneficial consequence of TRPC6 activation in ischemic stroke, its cell-specific roles in the brain, what is known about the involvement of TRPC6 in neurovascular coupling, and the potential therapeutic options for targeting TRPC6.

## 2. TRPC6

TRPC is a subfamily of TRP channels that are expressed in many cell types, including neurons [25,26]. These nonselective, cell membrane cation channels consist of seven members, TRPC 1–7, which depolarize cells via Na^+^ influx and also allow an influx of extracellular Ca^2+^, so as to regulate downstream cellular responses. Thus, this facilitates metabolism, membrane depolarization, gene expression, cell proliferation, and apoptosis [27]. Although all members are expressed in the brain and can promote nonselective Ca^2+^ entry, the spatial and temporal expression patterns of each are unclear. Based on structure–function relationships, the TRPC family is grouped into four subsets: TRPC1, TRPC2, TRPC3/6/7 and TRPC4/5. Besides functioning as homotetramers, combinations of different TRPC subunits can form heterotetrameric complexes, which may regulate responses to neuropeptides and neurotransmitters with different properties than homotetrameric TRPC channels [28]. For instance, TRPC6 has high sequence homology with TRPC3 and TRPC7 subunits, and may form a heterotetramer with TRPC3 [29].

**Figure 1:**
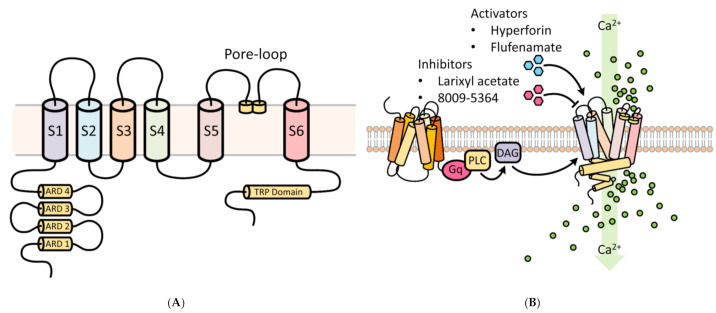
Functional aspects of transient receptor potential cation 6 (TRPC6) involved in Ca^2+^ cellular influx. (**A**) Structural features of the TRPC6 channel. TRPC6 possesses 6 membrane-spanning domains and 3 conserved domains, namely, a pore-loop motif, four NH2 terminal ankyrin repeat domains (ARD), and a COOH-terminal TRP box motif. The ARD domains participate in channel heterodimerization and trafficking, whereas the TRP domain regulates binding with the cytoskeleton and translocation to the cell surface. The pore-loop region (between the S5 and S6) is associated with an extracellular selectivity filter and an intracellular gate and allows for the passage of cations. ARD: ankyrin repeat domain; S1–S6 are the first to sixth transmembrane domains; TRP, transient receptor potential. (**B**) Regulators of TRPC6 channel activity. Receptor tyrosine kinases (RTKs) or G protein-coupled receptors (shown) increase TRPC6 activity by stimulating phospholipase C (PLC) to generate diacylglycerol (DAG). Several key experimental activators and inhibitors of TRPC6 are described in Section 6.

TRPC6 possesses three conserved domains, namely, a pore-loop motif, four NH2 terminal ankyrin repeat domains (ARD), and a COOH-terminal TRP box motif (Figure 1) [30]. The ARD domains are thought to participate in channel heterodimerization and trafficking, whereas the TRP domain may be important for regulating binding with the cytoskeleton and translocation to the cell surface [30]. The pore-loop region is associated with an extracellular selectivity filter and an intracellular gate. Different TRPCs exhibit different Ca^2+^ and Na^+^ permeability (P) ratios [31]. The P_Ca_/P_Na_ ratio of TRPC6 is 5, compared to 1.6 for TRPC3 [32,33].

TRPC6 activation mediates changes in cytosolic Ca^2+^, which govern diverse critical cellular functions (Figure 1), such as contraction, apoptosis, neuroprotection, angiogenesis, and cytokine production. TRPC6 and other TRPCs can be activated by phospholipase C (PLC) by numerous stimulations, such as inflammation and ischemia-reperfusion (IR) injury [34]. Through activation of G-protein coupled receptors (GPCRs) and receptor tyrosine kinases (RTKs), PLC can modulate TRPC channel activity by hydrolysis of phosphatidylinositol bisphosphate (PIP_2_) to diacylglycerol (DAG) and inositol trisphosphate (IP_3_) [35]. DAG activates TRPC3/6/7. IP_3_ causes Ca^2+^ release from internal stores, a process that triggers store-operated channel activation and may involve TRPC channels, although exactly how TRPC and store-operated channels interact is unclear [36,37]. Acting in a negative feedback manner, Ca^2+^ may reduce TRPC channel activity in synergy with protein kinase C (PKC), or via activation of calmodulin [38,39]. Additionally, TRPC3 and possibly TRPC6 may be activated as well in a β-arrestin-1-dependent manner [39,40,41]. These features make TRPC channels potential cellular sensors to respond to environmental changes by regulating intracellular Ca^2+^.

Structurally, TRPC6 is tethered directly to the cytoskeleton or extracellular matrix [42]. Studies on mouse embryonic fibroblasts and ventricular myocytes have suggested that TRPC6 may mediate the influx of Ca^2+^ in response to mechanical stress on the cell membrane [43,44]; however, TRPC6, expressed by various mammalian cell lines or in lipid bilayers, does not function as a mechanoreceptor [45,46]. Nonetheless, it may have evolved to discriminate different mechanical stimuli based on its interactions with the cytoskeleton or extracellular components. Therefore, TRPC6 may act as a secondary mechanoreceptor that contributes to the regulation of intracellular Ca^2+^ or depolarization of the membrane potential through inward Na^+^ and/or Ca^2+^ currents.

## 3. Cell-Specific Roles of TRPC6

**Figure 2:**
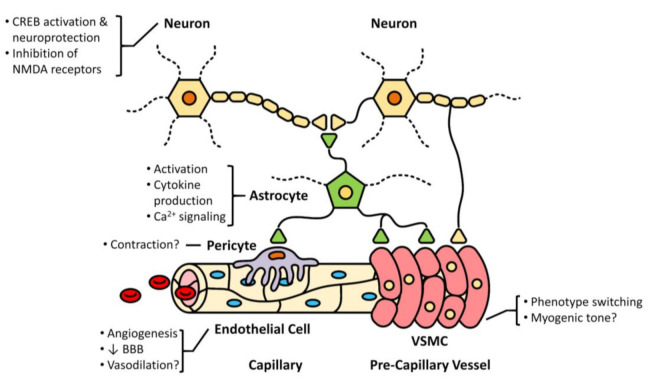
TRPC6 and the neurovascular unit regulating homeostatic cerebral blood flow. Depicted are the cellular constituents at the capillary and precapillary levels, as well interactions with neurons and astrocytes. The cell-specific roles of TRPC6 in the context of ischemic stroke, both established and conceptualized, are provided. There is evidence that TRPC6 is expressed in neurons, astrocytes, pericytes, endothelial cells, and vascular smooth muscle cells (VSMC). For more details refer to Section 3.

TRPC6 channels are expressed in neurons and other cells of the neurovascular unit (Figure 2). Their cell-specific role and importance to neurovascular coupling, however, requires further investigation.

### 3.1. Astrocytes

TRPC6 is detected in cultured astrocytes of rats [47,48]. Expression in primary cultures is regulated by glutamate. The NMDA receptor subtypes 2A (NR2A) and 2B (NR2B) are the major components of NR2 subunits of NMDA receptors in the adult brain [49]. NR2A containing NMDA receptors facilitate neuronal death, while blocking NR2B attenuates cell death after ischemic stroke [50,51]. Activation of NR2B-containing NMDA receptors increased TRPC6 degradation, whereas activating NR2A-containing receptors increased expression [48]. TRPC6 is also evident in mouse astrocytes, and the expression increased by treatment with interleukin (IL)-1β [52]. Additionally, it was established that TRPC6 is found in mouse neural stem cells. These undifferentiated precursor cells can differentiate into astrocytes, neurons, oligodendrocytes, and constitutionally express blue/red light-sensitive photoreceptors [53]. This study reported increased proliferation and astrocyte differentiation for mouse neural stem cells, under low power blue light, with TRPC6 being activated by Gq-coupled melanopsin (Opn4). Additionally, TRPC6 expression was detected in astrocytes of the optic nerve head in C57BL/6 mice [54] suggesting potential involvement in glaucoma pathogenesis [55].

Sphingosine-1-phosphate (S1P) plays a vital role in cell growth, survival, and migration [47,56]. Hisashi et al. demonstrated that S1P increases intracellular Ca^2+^ concentrations in astrocytes via activation of mitogen-activated protein kinase (MAPK) and TRPC6, resulting in the increased expression and release of chemokine (C-X-C motif) ligand 1 CXCL1 [47]. Beskina et al. demonstrated that TRPC6 is involved in IL-1β-induced Ca^2+^ signaling in mouse astrocytes. In this study, the expression of TRPC6 was increased in cortical astrocytes of mice treated with IL-1β, while receptor-operated Ca^2+^ entry was reduced with the knockout (KO) of the Trpc6 gene [52]. The authors suggested that disruption of Ca^2+^ homeostasis in astrocytes by chronic IL-1β treatment, due to increased TRPC6 activity, might ultimately lead to neurodegenerative diseases.

### 3.2. Neurons

TRPC6 is expressed in many different brain regions, such as the cortex, hippocampus, cerebellum, basal ganglia, thalamus, hypothalamus, and dorsal root ganglia [57]. Numerous studies have documented that the expression of TRPC6 in primary cultured neurons or neuronal cell lines is protective under various stress conditions [48,58,59,60,61,62].

Ca^2+^ is a ubiquitous second messenger that affects neuron proliferation and survival in brain development. Ca^2+^ signals also influence differentiation, dendrite morphology, and axon guidance through actions on cytoskeletal dynamics and cell adhesion [63]. Each neuron expresses a unique set of Ca^2+^-permeable channels, which allows for the generation of intracellular Ca^2+^ signals of a particular time course, amplitude, and location [64]. TRPC-meditated Ca^2+^ influx is involved in nerve-growth-cone guidance, synapse formation, synaptic transmission, neuronal survival, and sensory transduction, downstream of RTKs or GPCRs that are expressed by the neuron [65]. Brain-derived neurotrophic factor (BDNF) is a pro-survival neuronal peptide that is important during brain development. BDNF binds to the receptor tropomyosin receptor kinase B (TrkB), to activate the protein kinase B (Akt) and adenosine 3’,5’-cyclic monophosphate (cAMP) response element-binding protein (CREB) pathways, to promote survival [66]. Downregulation of TRPC6 prevents the protective effect of BDNF on granule cells, leading to apoptosis [67]. Ca^2+^ influx through TRPC6 activates Ca^2+^/calmodulin-dependent protein kinase (CaMK) and MAPK to phosphorylate CREB, an important transcription factor, leading to neuronal survival [63].

### 3.3. Endothelial Cells

TRPC6 is differentially expressed in endothelial cells from different vascular beds and participates in a diverse range of endothelium-related functions, such as control of vascular tone, regulation of vascular permeability, angiogenesis and remodeling, and apoptosis. In the brain vasculature, TRPC6 is found in mouse microvascular endothelial cells and human cerebral artery endothelial cells [68,69,70].

One of the most crucial functions of the endothelium is maintaining BBB integrity. Several studies have implicated TRPC6 as essential in the regulation of endothelial permeability. Singh et al. reported that Gα_q_ activation of TRPC6 induced Ca^2+^ entry and activation of Ras homolog family member A (RhoA), which resulted in myosin light chain-dependent endothelial cell shape changes and increased gap formation between endothelial cells [71]. The net effect was the increased permeability of endothelial monolayers. One pathological outcome is lung edema after ischemic-reperfusion injury from TRPC6 recruitment to caveolae of endothelial cells in rats and mice, thereby permitting Ca^2+^ influx [72,73]. Additionally, Cheng et al. reported that heteromeric TRPC6 is involved in vascular endothelial growth factor (VEGF)-induced Ca^2+^ influx in human microvascular endothelial cells [74]. This group subsequently demonstrated that VEGF-induced extracellular Ca^2+^ entry, proliferation, and tube formation are attenuated in human microvascular endothelial cells that overexpress a dominant-negative TRPC6 mutant [75]. Further research is needed to establish what role, if any, TRPC6 plays in endothelial function and BBB integrity in ischemic stroke. Conceivably, activation of TRPC6 in endothelial cells could lead to vasodilation by various means, including the stimulation of nitric oxide synthase 3 (NOS3) [37], but whether this occurs in the cerebral vasculature has not been reported. Excessive cytosolic Ca^2+^ increases would lead to endothelial cell injury or cell death, leading to loss of gap junctions and increased permeability, thus contributing to cerebral edema and swelling of the brain in ischemic stroke. The increase in cerebrospinal fluid pressure would further restrict blood flow to the penumbra, expanding the infarct in the area at risk.

### 3.4. Vascular Smooth Muscle Cells (VSMCs)

Activation of TRPC6 in VSMCs of the cerebral vasculature might contribute to the no-reflow phenomena and attenuation of metabolic vasodilation in ischemia. Two recent studies documented that TRPC6 in VSMCs contributes to pressure-induced constriction of cerebral arteries [76,77]. This is an intrinsic characteristic of small arteries and arterioles to constrict in response to increases in intraluminal pressure. Details on exactly how TRPC6 in VSMCs couples to constriction by increasing intracellular Ca^2+^ are unclear, with some suggestion that it may function, not in the plasma membrane, but rather as a “downstream signal amplifier” mediated via PLC, DAG, cytochrome P450 omega-hydroxylase and 20-Hydroxyeicosatetraenoic acid (HETE) [76,78].

## 4. Neurovascular Coupling in Ischemic Stroke

In ischemic stroke, the dysfunction of the cells in the neurovascular unit and the communication between them characterizes the impaired neurovascular coupling [79,80], which normally acts to compensate for the tissue hypoxia and release of metabolic vasodilators [81,82]. Astrocytes and propagation of hyperpolarization via endothelial cells are a major component of neurovascular coupling, and astrogliosis or reactive astrocytosis is found around cerebral vessels following ischemic stroke [83]. Whether this affects the function of astrocytes to modulate cerebral blood flow via neurovascular coupling is unclear. Ischemic stroke induces cortical spreading depressions (CSDs), which are slowly propagating waves of sustained depolarization of neurons and glial cells. CSDs characterized by injured astrocytes can lead to impaired neurovascular coupling in ischemic stroke [84]. Additionally, in ischemic stroke, astrocytes release 20-HETE to induce vasoconstriction, limiting cerebral blood flow and contributing to the no-reflow phenomena [84,85].

Notably, the vascular response to direct smooth muscle vasodilators is unaffected by ischemic stroke, suggesting that dysfunction in other cell types in cerebral vessels accounts for the impaired neurovascular coupling [86]. However, TRPC6 has been implicated in VSMC phenotype switching under ischemic conditions [87,88]. TRPC6, along with other TRP channels subfamilies, are expressed in pericytes [89]. It is not clear whether capillary contraction in pericytes is directly mediated by TRPC6. However, pericytes regulate capillary diameter to control cerebral blood flow, constrict in response to hypoxia in ischemic stroke and die in rigor. This results in a long-lasting increase in capillary resistance that worsens cerebral ischemic injury after stroke [90]. Loss of pericytes may also exert adverse effects on neurotropic-dependent neuronal survival [84], which could contribute to neurovascular coupling dysfunction. Conversely, cerebral ischemia can induce metalloproteinase-9 (MMP-9) activation and release in pericytes, which promotes long-term capillary damage and tight-junction breakdown [91]. Taken together, preventing pericyte dysfunction may be a promising therapeutic target for the treatment of ischemic stroke.

Caveolae-mediated transcytosis in endothelial cells, which is suppressed under normal conditions to maintain BBB integrity [92], is activated following ischemic stroke and may contribute to neuroinflammation [84]. On the other hand, the caveolae of arteriolar endothelial cells (aECs) may play an active role in neurovascular coupling and vasodilation by relaying signals from neurons to cerebral smooth muscle cells [93]. Thus, misdirected trafficking of caveolae-dependent endothelial cell communication may underlie the pathology of ischemic stroke. Notably, an intact capillary–arteriole continuum via endothelial cell junctions is a necessary feature of the process, whereby capillary endothelial cells sense increased extracellular K^+^ from neuronal cell release (due to hypoxia/ischemia) and initiate retrograde hyperpolarization to increase local cerebral blood flow [94]. This process might be impaired by increased Ca^2+^ entry into endothelial cells via TRPC6 activation.

## 5. Role of Neuronal TRPC 6 in Ischemic Stroke

TRPC6 is highly expressed in the central nervous system and is important in neuronal development and survival [95]. The overactivation of NMDA receptors occurs after an ischemic stroke due to excessive glutamate-mediated calpain stimulation, which promotes proteolysis of TPRC6 [96]. Degradation of TRPC6 contributes to neuronal death via downregulation of the transcription factor CREB, leading to neuron apoptosis [22,97]. In contrast, inhibition of TRPC6 degradation promotes neuron survival, reduces infarct size, and improves behavioral performance [22]. Furthermore, a TRPC6 activator was found to ameliorate neuronal death in ischemic stroke and this was associated with improved phosphorylated CREB (p-CREB) activity [96]. As noted, the combination treatment of oxiracetam and bone marrow stromal cells increases TRPC6 and p-CREB levels and protects from neuronal death in ischemic stroke [98].

Increased degradation of TRPC6 after ischemic stroke may result in neuronal damage. Activating TRPC6 blocked neuronal death, while inhibiting TRPC6 degradation via a fusion peptide based on the calpain cleavage site (TAT-C6 peptide), reduced infarct size and improved behavioral performance through the CREB signaling pathway [22]. Enhanced TRPC6 expression suppressed NMDA receptor-mediated Ca^2+^ neurotoxicity, and several molecular probes modulated brain function and promoted neuroprotection and recovery in ischemic stroke by enhancing TRPC6 channel function [20]. Notably, infarct volume in TRPC6 transgenic mice was less than in their wild type littermates, with the transgenic mice exhibiting better behavior performance and lower mortality [20]. It was proposed that TRPC6 might regulate the phosphorylation of NMDA receptors, thereby attenuating their activity.

The immunological pathway is also involved in TRPC6 activity. A study in mice suggests that the deleterious effects of the pro-inflammatory cytokine IL-17A in cerebral IR injury, involves—in part—the degradation of TRPC6 [99]. IL-17A KO or anti-IL-17A monoclonal antibody attenuated activation of calpain 3 days after reperfusion, while recombinant IL-17A increased its activation and IR injury. Brain injury and neurological deficits were primarily abolished by genetic KO of IL-17A, an IL-17A antibody, or a calpain inhibitor. Moreover, the calpain-specific inhibitor increased TRPC6 expression.

On the contrary, TPRC6 expression levels may increase following ischemic stroke, leading to Ca^2+^ overload that contributes to neuronal death [61]. Indeed, TRPC6 elevation was reported to promote Na^+^ influx, which induces membrane depolarization and activation of NMDA receptors and Ca^2+^ influx. In this study, deletion of TPRC6 attenuated Ca^2+^ overload-induced neurotoxicity in ischemic stroke [61]. Another group also reported that loss of TRPC6, together with TRPC3 and TRPC7, is neuroprotective [100]. KO of TRPC3/6/7 in a mouse cerebral IR model had an anti-apoptotic effect on astrocytes, resulting in reduced infarct volume and neurological deficits [101]. The conflicting results of these studies with a neuroprotective role for TRPC6 may be related to the animal model used, duration of a stroke, and confounding contribution of other TRPC isoforms, as well as potentially to the choice of anesthesia [102]. Initially, TRPC6 may increase Ca^2+^ in endothelial cells and cause NO generation to compensate for the occlusion and to reduce the size of the area at risk. With prolonged ischemia, the endothelial cells will die, and this compensation will be lost. In neurons, TRPC6 may stimulate protection at first, but may contribute to Ca^2+^ overload and injury in the long term.

Interestingly, an imbalance in TRPC6 expression (too much or too little) may be associated with neuronal death in ischemic stroke. Alterations in TRPC6 activity following ischemic stroke is likely to alter intracellular Ca^2+^, that modulates NMDA receptor activity or affects the release of vasoactive mediators, such as nitric oxide (NO) and AA derivatives. Currently unknown, is whether TPRC6 has a role in the abnormalities of neurovascular coupling in ischemic stroke. The activity of neurovascular coupling could be affected under these conditions due to the change in TRPC6 activities. As discussed, activation of TRPC6 in endothelial cells may compromise the integrity of the BBB.

Astrocytes in the ischemic areas are activated after ischemic stroke. These active astrocytes exert both beneficial and harmful effects on neurons in the ischemic area [103]. It is reported that TRPC6 leads to impaired Ca^2+^ homeostasis and exacerbates mitochondrial dysfunction and endoplasmic reticulum stress so as to promote cellular apoptosis [104], but whether this occurs in activated astrocytes is unclear. There is evidence that astrocytes protect brain tissue after ischemic stroke and reduce the occurrence of disability [22]. They also play an essential role in neural network reconstruction. Studies in mouse models of cerebral ischemia have also shown that astrocytes can transfer into immature neurons after ischemia. Blocking Notch signaling in astrocytes could initiate this process [105]. An earlier study reported that TRPC6 has an essential role in regulating neural stem cell proliferation and differentiation [53]. Unfortunately, whether it is evolved in regulating the transformation of astrocytes into neurons is still unknown.

## 6. Therapeutic Opportunities

A number of compounds that either activate or inhibit TRPC6 activity have been investigated in biomedical research [106]. Some are discussed here (Figure 1). St John’s wort contains a component, hyperforin, a protonophore, that indirectly activates TRPC6 [107,108] by inhibiting TRPC6 breakdown and attenuates brain damage from transient middle cerebral artery occlusion (MCAO) in rats [96]. Another molecule, the nutritive polyphenol “resveratrol”, when applied for 7 days before MCAO onset, has neuroprotective effects by activating the TRPC6/CREB pathway and decreasing calpain activity [109]. Similarly, neuroprotection in a rat stroke model was observed via TRPC6/CREB using “Neuroprotectin D1” when applied after reperfusion [110]. The predominant constituent of green tea, (-)-epigallocatechin-3-gallate (EGCG), introduced immediately after ischemia, demonstrates neuroprotection by decreasing calpain activity and activating TRPC6/CREB via the mitogen-activated protein kinase (MEK)/extracellular pathway [111]. Furthermore, “calycosin”, a major isoflavonoid in Radix Astragali Mongolici, protected against ischemia-induced damage by inhibiting calpain activation and increasing TRPC6 and p-CREB levels [59]. In these studies, the TRPC6-CaMK-CREB pathway is common in mediating the actions of TRPC6 manipulations. Thus, TRPC6/CREB may be crucial in neuronal survival and a potential novel therapeutic target [112,113].

Recent studies have highlighted the benefit of stem cells in stroke recovery [114]. The role of TRPC6 in bone marrow stromal cell (BMSC) transplant was investigated, where the application of BMSCs overexpressing TRPC6 reduced brain injury in a rat IR model [115]. This was associated with synergistic activation and increased levels of TRPC6 in brain tissue, ostensibly due to enhanced production of BDNF, a well-known protective neurotrophic factor. Evidence indicated that decreased neuronal death was due to the TRPC6/CREB pathway and inhibition of calpain activity, a common pathway associated with neuronal survival. A related study with this model demonstrated that BMSCs activate TRPC6 protective p-CREB signaling. Combined therapy of BMSCs with oxiracetam, which inhibited abnormal degradation of TRPC6 by decreasing the activity of calpain, resulted in significantly improved functional restoration and reduced infarct size [98].

The latest research shows potential for new TRPC6 agonists and antagonists in preclinical experiments, specifically related to neuroscience, which may have a useful role in ischemic stroke in the future. Tetrahydrohyperforin (IDN5706), a derivative of hyperforin and TRPC3/6/7 activator, was neuroprotective in a mouse model of Alzheimer’s disease by negating inhibitory effects of Aβ oligomers in hippocampal tissue [116]. Ladecola et al. showed that IDN5706 might have potential in vascular dementia, where targeting Aβ oligomers may delay cognitive impairment [117].

TRPC6-PAM-C20 is a selective TRPC6 modulator. When tested with another TRPC6 activator OAG (1-oleoyl-1-acetyl-sn-glycerol), there was a synergistic, robust increase in Ca^2+^ in human platelets [118]. Thus, this agent may have the potential to modulate platelet function before or after ischemic stroke. There is some evidence indicating that TRPC6 plays a critical role in platelet function via receptor-operated calcium (ROC) [119,120] or store-operated calcium (SOC) entry [121]. On the contrary, one report suggests Ca^2+^ influx via TRPC6 may not have functional relevance in platelet hemostasis [122]. Whether TRPC6 is a potential target for antithrombotic therapy requires further investigation. Another TRPC6 activator, flufenamic acid modulated GABAa receptors, quashing epileptiform activity in the hippocampus [123]. Other TRPC3/6 activators, such as GSK1702934A, transiently increased the perfusion pressure of isolated rat hearts retrogradely perfused via aortic cannulation, an effect blocked by pretreatment with the TRPC3/6 blocker GSK2293017A [124]. OptoBI-1, a derivative of GSK1702934A, is a light-sensitive TRPC3/6/7 agonist that was found to suppress hippocampal action potential firing [125]. Small compound derivatives of piperazine, PPZ1 and PPZ2, are TRPC3/6/7 agonists that can induce BDNF-like neurite growth and neuroprotection in cultured neurons by triggering Ca^2+^ dependent CREB signaling [126]. Thus, PPZ1 and PPZ2 represent potential agents for post-stroke recovery.

Inhibitors of TRPC6, such as larixyl acetate [127] and GsMTx4 [128], have been shown to have protective actions against traumatic brain injury and myocardial infarction. Synthetic 20-HETE has TRPC6 activation effects, whereas blocking 20-HETE formation with N-hydroxy-N’-(4-n-butyl-2-methylphenyl)formamidine (HET0016) reduced cerebral infarction volume and improved cerebral blood flow in a pediatric-rat pup cardiac asphyxia model [129] and MCAO in adult rats [130]. SAR7334 and the tryptoline derivative 8009-5364 are other highly specific TRPC6 antagonists that diminished acute hypoxia-induced vasoconstriction in the lung [131]. Their activity in the brain is yet to be thoroughly tested but they may be useful during subarachnoid hemorrhage (SAH)-related vasoconstriction damage. Similarly, BI 749327, although not investigated in the brain, has a cardio/renal protective effect by reducing fibrosis [132].

So far, preclinical safety tests have not been completed on any of these agents and none have been approved for clinical trials in humans. Thus, their potential clinical efficacy cannot be asserted definitively. However, their potential benefit against damage from cerebral ischemia warrants further investigation.

## 7. Conclusions and Clinical Perspectives

Stroke care is limited due to the lack of effective primary or good secondary preventative agents that can control the excitotoxic damage that results from ischemic infarction. In addition, there is a definite need for medications that can help in stroke recovery. Various pathways discussed in this review that involve TRPC6 can provide neuroprotection and assist post-stroke recovery. Several small molecules targeting TRPC6 have been shown to demonstrate potential benefits in animal experiments. Many of these molecules have the potential to be translated into human clinical trials. However, there is further need for studies that could help determine the mechanisms underlying these benefits. Additional knowledge on the cell-specific role of TRPC6, utilizing cell-specific KOs of TRPC6, as well as information on the implications of its involvement in neurovascular coupling, is critically needed. Whether targeting TRPC6 alone or as a combination of various TRPC channels will mitigate the harm of stroke and enhance recovery, only time and science will tell.
